# Neurochemical mechanism of muscular pain: Insight from the study on delayed onset muscle soreness

**DOI:** 10.1186/s12576-023-00896-y

**Published:** 2024-01-24

**Authors:** Kazue Mizumura, Toru Taguchi

**Affiliations:** 1 Nagoya University, Nagoya, 464-8601 Japan; 2 Department of Physiology, Nihon University School of Dentistry, 1-8-13 Kanda-Surugadai, Chiyoda-ku, Tokyo, 101-8310 Japan; 3 Department of Physical Therapy, Faculty of Rehabilitation, Niigata University of Health and Welfare, Niigata, 950-3198 Japan; 4 Institute for Human Movement and Medical Sciences (IHMMS), Niigata University of Health and Welfare, Niigata, 950-3198 Japan

**Keywords:** Delayed onset muscle soreness, Lengthening contraction, Nerve growth factor, Glial cell line-derived neurotrophic factor, Mechanical hyperalgesia, Muscle pain

## Abstract

We reviewed fundamental studies on muscular pain, encompassing the characteristics of primary afferent fibers and neurons, spinal and thalamic projections, several muscular pain models, and possible neurochemical mechanisms of muscle pain. Most parts of this review were based on data obtained from animal experiments, and some researches on humans were also introduced. We focused on delayed-onset muscle soreness (DOMS) induced by lengthening contractions (LC), suitable for studying myofascial pain syndromes. The muscular mechanical withdrawal threshold (MMWT) decreased 1–3 days after LC in rats. Changing the speed and range of stretching showed that muscle injury seldom occurred, except in extreme conditions, and that DOMS occurred in parameters without muscle damage. The B2 bradykinin receptor—nerve growth factor (NGF) route and COX-2—glial cell line-derived neurotrophic factor (GDNF) route were involved in the development of DOMS. The interactions between these routes occurred at two levels. A repeated-bout effect was observed in MMWT and NGF upregulation, and this study showed that adaptation possibly occurred before B2 bradykinin receptor activation. We have also briefly discussed the prevention and treatment of DOMS.

## Introduction

The muscle is the largest organ in the body, occupying 40% or more of the body weight, and is always exposed to wear and tear during daily activities; however, pain or tenderness from this tissue has not been shed much light on by physicians and orthopedists. Since no one dies from muscle pain, since many researchers believe that the pain mechanism for superficial tissue (skin) and deep tissue, including muscles, must be the same, and since muscle pain has been handled mainly and effectively by traditional medicine (acupuncture, massage, and herbal medicine), its mechanism has not been studied thoroughly. Since the 1980s, pain mechanisms have been intensively studied and brought new discoveries, mainly on cutaneous pain, and many people are suffering from deep pain such as chronic low back pain, myofascial pain syndromes, and delayed-onset muscle soreness (DOMS), medical/physiology textbooks still devote only minimal pages to neurochemical mechanisms of pain and almost no pages to muscle pain.

The characteristics of muscle pain are: (1) diffuse aching, (2) often referring to a distant somatic area [1], and (3) often accompanied by changes in muscle hardness [2, 3]. Related to these characteristics, nociceptive inputs from the muscle (1) have stronger influences on spinal neurons than cutaneous inputs [4], (2) are under the stronger influence of the descending inhibitory system [5], and (3) nerve injury has a stronger influence on muscular afferents [6, 7].

These characteristics differentiate muscle pain from cutaneous pain. Thus, muscle pain must be studied independently.

## Historical overview of basic studies on muscle pain

Sensory afferents responsible for conveying noxious signals from the muscle to the spinal cord were initially identified by Paintal in 1960 [8]. These afferents were found to respond to muscle pressure and were transmitted via Aδ- (Group III) fibers. The prevailing belief has been that muscle pain primarily conveyed through group III (Aδ) and group IV (C) fibers [9]. However, an alternative perspective was proposed by Weerakkody et al. suggesting that the sensation of muscle soreness following exercise is attributed not to nociceptors but rather to large fiber mechanoreceptors (muscle spindles and tendon afferents) [10]. Neonatally capsaicin-treated rats, in which C-fibers were destroyed, showed no decrease in the muscular mechanical withdrawal threshold (MMWT) (corresponding to muscular mechanical hyperalgesia in humans) after lengthening contraction (LC), showing that C-fibers, not thick A-fibers, are responsible for DOMS [11]. Vibrations that efficiently excite muscle spindles do not induce pain [12], but rather reduce acute and chronic muscle pain [13]. We found many drawbacks in Weerakkody’s reports and did not refer to them further.

Muscle afferents that respond to algesic substances and are transmitted by C- (Group IV) fibers have been reported by Mense and Schmidt [14]. Kumazawa and Mizumura [15] reported polymodal receptors that respond to all forms of algesic stimulation, that is, mechanical stimulation, heat and algesic substances, and with both Aδ- and C-fibers. Sensitization to heat has also been observed, similar to cutaneous afferents [15]. The existence of thin-fiber afferents that are insensitive to mechanical stimulation, but become sensitive after the induction of inflammation or repetitive stimulation (mechanically insensitive nociceptors), has been reported in the knee joint [16] and skin [17]. Similar receptors have been reported in muscles in an abstract form [18].

**Fig. 1:**
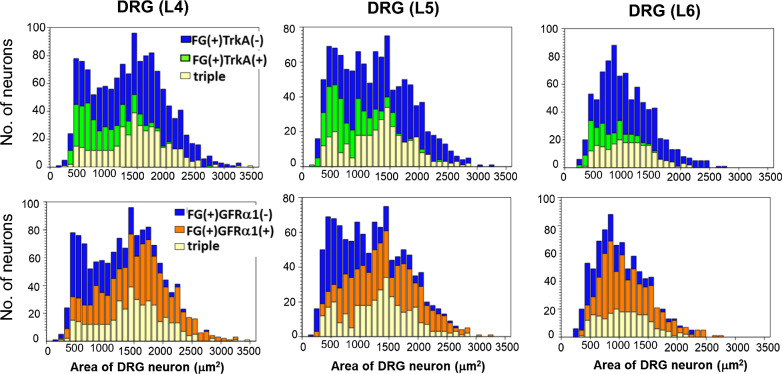
Size distribution of muscle innervating DRG neurons and expression of NGF and GDNF receptors. Muscle-innervating DRG neurons were traced by the retrograde transport of Fluorogold (FG). Upper column: Blue, FG^+^ and TrkA^−^ neurons; green, FG^+^/TrkA^+^ neurons; yellow, triple positive neurons (FG^+^/TrkA^+^/GFRα1^+^). Lower column: blue, FG^+^/GFRα1^−^ neurons; orange, FG^+^/GFRα1^+^ neurons: yellow, triple positive neurons (FG^+^/TrkA^+^/GFRα1^+^). Numbers of cells expressing TrkA, GFRα1 and both in each size range are cumulatively presented in each bar. The addition of all three in each size range provided the number of cells innervating the GC in that size range. Modified from Murase et al. [19]

The size and segmental distribution of muscle primary afferent neurons (dorsal root ganglion (DRG) neurons) are quite different from cutaneous neurons [19]. The size distribution of the skin-innervating DRG neurons or DRG neurons of the mixed nerve is largest in the small size range (skewed in small-sized range) and monotonically decreases to medium and large size ranges [20–22]. In contrast, the DRG neurons innervating the gastrocnemius muscle (GC) lack this skewing of the population toward smaller cell sizes, and the histogram tends to be symmetrical and dome-shaped (Fig. 1) [19]. Similar observations have been reported in previous studies [21, 23, 24]. Muscle DRG neurons expressing tropomyosin-related kinase A (TrkA), which is a high-affinity receptor for nerve growth factor (NGF) and is thought to be expressed in nociceptors [20, 21], are widely distributed from small to large sizes (Fig. 1 upper lane). Muscle DRG neurons with thin-axons might have larger cell bodies [25, 26]. As reported for the mesencephalic trigeminal nucleus [27, 28], where trigeminal proprioceptive afferent neurons are located, the large TrkA-positive neurons in Fig. 1 might include proprioceptive afferents. However, the role of NGF in these neurons in adult animals remains unknown.

Spinal projections of electrophysiologically identified single-muscle C-fibers were traced [29] using an intracellular injection of PHA-L in female guinea pigs. Labelled axons project two or three segments craniocaudally and give off collaterals in laminae I and II, where nociceptors synapse to secondary neurons. The central collaterals of muscle afferents are less dense than the cutaneous ones [30] but denser than the visceral collaterals [31, 32], and the segmental distribution is wider than the cutaneous ones but less wider than the visceral ones. A wider segmental distribution than cutaneous ones might be related to the less localized nature of muscle pain.

In cats, thalamic neurons excited by electrical stimulation of the gastrocnemius nerve are distributed not in the midst of the ventral posterolateral nucleus, but rather in its periphery [33]. Similar projections of cutaneous afferents have been also reported [34]. Recently, Todd et al. reported that 95% of the lamina I neurons of the lumbar spinal cord (but a lower % from the cervical cord) project to the lateral parabrachial nucleus [35], not to the thalamus, in rats. The percentage of neurons projecting to the lateral parabrachial nucleus is lower in cats than in rats. Whether this is true for lamina I neurons with muscular inputs remains unknown. However, this point requires further clarification.

Several pain models have been developed to study pathophysiological mechanisms underlying muscle pain. Inflammation model induced by carrageenan or complete Freund’s adjuvant has been well studied, and sensitization of Group III and IV afferents by inflammatory mediators such as tumor necrosis factor (TNF-α [36], NGF [37], glial cell line-derived neurotrophic factor (GDNF) [38], and protons [39] has been reported. However, inflammation is not often observed in painful muscle conditions; thus, other models are required. Sluka et al. [40] developed an acid injection model by injecting acidic saline at pH4–6 two times at intervals of 5 days, resulting in long-lasting bilateral mechanical hyperalgesia without inflammation. A repeated (intermittent) cold stress model was used for fibromyalgia model [41–44]. The prevalence of myofascial pain syndrome, which is characterized by trigger point-like sensitive spots and muscle hardening (taut band) in the muscle, has been notably high [45]. Delayed onset muscle soreness (DOMS) has been reported to exhibit muscle changes akin to those observed in myofascial pain syndrome [3, 46, 47]; thus, it has been used in the study on myofascial pain syndrome. Intramuscular injection of NGF has also been used as a model of muscle pain. It induces muscle mechanical hyperalgesia and referred pain; thus, it can be used to study myofascial pain syndrome. Because NGF has been used in clinical settings [48–50], its safety for human use is assured. Thus, NGF became one of the few powerful tools for experimental study of muscle pain in humans [51–54]. Recently, a model of craniofacial myalgia, represented by myogenous temporomandibular disorder and tension-type headache, was developed by electrically contracting the masseter muscles and sensitizing the cervical muscles (trapezius muscle) with NGF [55]. It develops only in female rats, which is similar to the higher incidence of temporomandibular disorder and tension-type headaches in female humans.

In the following section, we focus on the mechanism of DOMS, which we have been studying in animals for approximately 20 years.

## Brief overview of studies on LC-induced changes including DOMS

DOMS is a common experience shared by nearly everyone, often occurring after activities such as mountain climbing for the first time, an occasional baseball game, or running in a once-a-year school sports meeting. Initially described by Hough in 1902, DOMS has been a recurrent sensation in various individuals [56]. DOMS is characterized by tenderness and movement related pain, with usually no pain at rest [57]. This is a common consequence of unaccustomed strenuous exercise. Athletes who exercise daily experience DOMS when performing different types of sports or practicing new skills. DOMS is different from the acute pain experienced during and shortly after exercise, and it typically appears after some pain-free period (12–24 h), peaks at 24–72 h, and disappears within 7 days after exercise without any medical treatment [57–59]. Exercise which induces DOMS has been identified to be eccentric type (lengthening contraction, LC) [60], where muscle is being stretched while it is contracted. LC has been a fascinating research topic in sports and athletes from both theoretical and practical points of view because it can produce higher force with less energy (O_2_ consumption) than shortening contraction, and training with LC increases muscle strength and size compared to shortening contraction [61–63]. A possible mechanism for LC to produce a higher force than shortening contraction can be found in the literature [64, 65]. Fast-twitch fibers (myosin heavy chain IIb and IIx) are more susceptible to DOMS than slow muscles [66].

LC is usually accompanied by not only DOMS but also reduces the maximum power of the muscle and range of motion [67–69]. These changes have been considered to be related to damage to the subcellular structure of muscle fibers: focal Z-band disruption (streaming or smearing), focal sarcomere disruption, organelle displacement, and cytoskeletal disruption [70, 71]. While severe muscle damage has been reported in animals, the existence and extent of such damage in humans have been subjected to dispute and contention. Crameri et al. showed that muscle damage is induced only when the muscle is electrically stimulated to induce LC but not voluntarily contracted in humans [72], while Lauritzen et al. showed that even voluntary LC can induce muscle fiber damage when LC is performed with maximal power [73]. Leakage of creatinine kinase into the blood is also considered to be related to microdamage of the sarcolemmal membrane. Because fast twitch fibers (myosin heavy chain IIb and IIx) are more susceptible to DOMS than slow muscles [66], depletion of the energy source could be the cause of damage. Biochemical and mechanical factors, including cytoskeletal changes, have been examined (see [74] for a review); however, the details of these changes are still unclear.

Presently, the most popular view of the DOMS mechanism is the microdamage of muscle fibers and subsequent inflammation (mononuclear cell infiltration, macrophage accumulation, etc.). However, the changes considered indicative of muscle damage did not parallel the time course and severity of DOMS [75]. There are also reports that inflammatory cell infiltration into muscle fibers does not significantly induce soreness after voluntary contractions in humans [76, 77].

## Neurochemical mechanism of delayed onset muscle soreness—our findings

### DOMS model in rats and mice

**Fig. 2:**
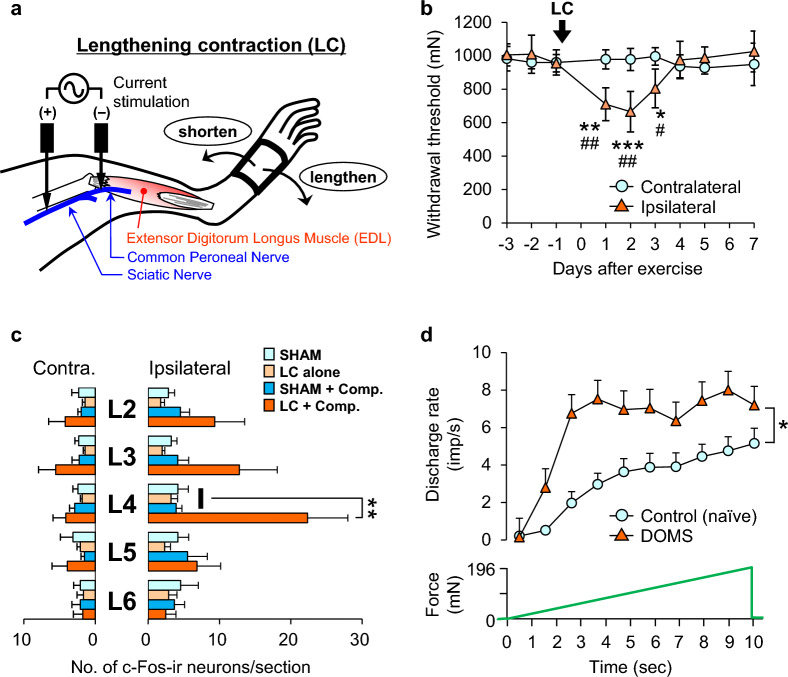
A rat model of DOMS. **a**: Method applying lengthening contractions mainly to the extensor digitorum longus (EDL) muscle. One pair of insulated needle electrodes, except for their tips, was inserted near the common peroneal and sciatic nerves. While the muscle was contracted by electrical stimulation (50 Hz for 1 s), it was stretched. This was repeated 500 times with **a** 3 s resting period. **b**: Change in withdrawal threshold after lengthening contraction (LC). The withdrawal threshold decreased for three days after LC. **c**: c-Fos expression in the superficial dorsal horn. A significant increase in the number of c-Fos-positive neurons was observed only when the muscle was compressed after LC at L4, where the nerve innervating the EDL terminated. **d**: Time course of discharges of muscle thin-fibers in response to ramp mechanical stimulation (0–196 mN in 10 s, lower-most trace). The discharge rate was significantly higher in the fibers recorded from rats two days after LC (orange triangles) than in those recorded from control animals (blue circles). **a** and **c**: Modified from Taguchi et al. [78]; **b**: Modified from Taguchi et al. [147]; **d**: Modified from Taguchi et al. [83]

Many animal studies have been performed using downhill treadmills running all-out. With this method, systemic effects could not be denied; therefore, we employed a one-hindleg exercise in which we electrically stimulated the common peroneal nerve innervating the extensor digitorum longus muscle (EDL) with the use of insulated acupuncture needles except tips. Using this method, we avoided directly stimulating the muscle because it has been reported to induce more muscle damage (Crameri et al. [72], also see the previous section). Simultaneously, the lower hind leg was stretched using a motor (Fig. 2a) [78]. The gastrocnemius muscle can be used as a target muscle by changing the nerve to stimulate and the direction of ankle movement [79]. Following the completion of lengthening contractions (LC), the muscular mechanical withdrawal threshold (MMWT) was assesses using a Randall-Selitto apparatus equipped with a cone-shaped probe (diameter of the base: 9 mm, tip: 2.6 mm) exhibited a decrease 1 day after the LC. This decrease persisted for up to 3 days post LC and subsequently returned to the pre-exercise level by the fourth day (Fig. 2b). The probe used was confirmed to measure the deep MMWT both experimentally [41, 80] and theoretically [81]. However, the cutaneous mechanical withdrawal threshold did not change [78]. The time course of DOMS is similar to that in humans. To ascertain whether the decrease in the MMWT represented muscular nociception, we examined c-Fos expression in the superficial layers (laminae I–II) of the lumbar dorsal horn, where nociceptive transmission occurs. An increase in c-Fos expression was not observed in animals receiving only stretching (SHAM), compression, or LC. Animals with muscle compression 2 days after LC showed increased c-Fos expression in the superficial dorsal horn at L4, where the nerve innervating the lower hind leg extensors mainly terminates (Fig. 2c). These results align with the fact that DOMS is usually not accompanied by spontaneous pain [57], and the muscle after LC is tender.

To further validate these observations, we recorded ex vivo the mechanical responses of muscular thin-fiber afferents (mainly C-fibers), which transmit noxious information from the muscle [15, 82]. The magnitude of the mechanical response (number of discharges induced) to ramp-shaped pressure stimulation increased, and the response threshold decreased in fibers recorded 2 days after LC (Fig. 2d) [83]. The sensitivity to heat, bradykinin, protons [83] and hypertonic saline [84] was not changed after LC. Spontaneous activity did not differ between the fibers recorded from the control and LC animals; this is considered to correspond to the absence of spontaneous pain in DOMS.

Existence of taut band-like hardening and trigger point-like sensitive spots in the exercised muscle has been reported [3, 47, 85]. DOMS usually disappears within a week after LC; however, it can become chronic (extended by one week after the end of LC) when LC is repeated daily for 2 weeks [85]. These observations support the idea that DOMS can be used as a model for mechanistic studies on myofascial pain syndrome.

Different types of knockout mice have been used to examine the involvement of receptor channels in DOMS. For this purpose, a mouse model of DOMS was developed using electrical stimulation of the tibial nerve [86] (details are introduced in the Section "Ion channels involved in mechanical sensitization in DOMS").

### Stretch speed and range of motion, and DOMS and histological changes of the muscle

**Fig. 3:**
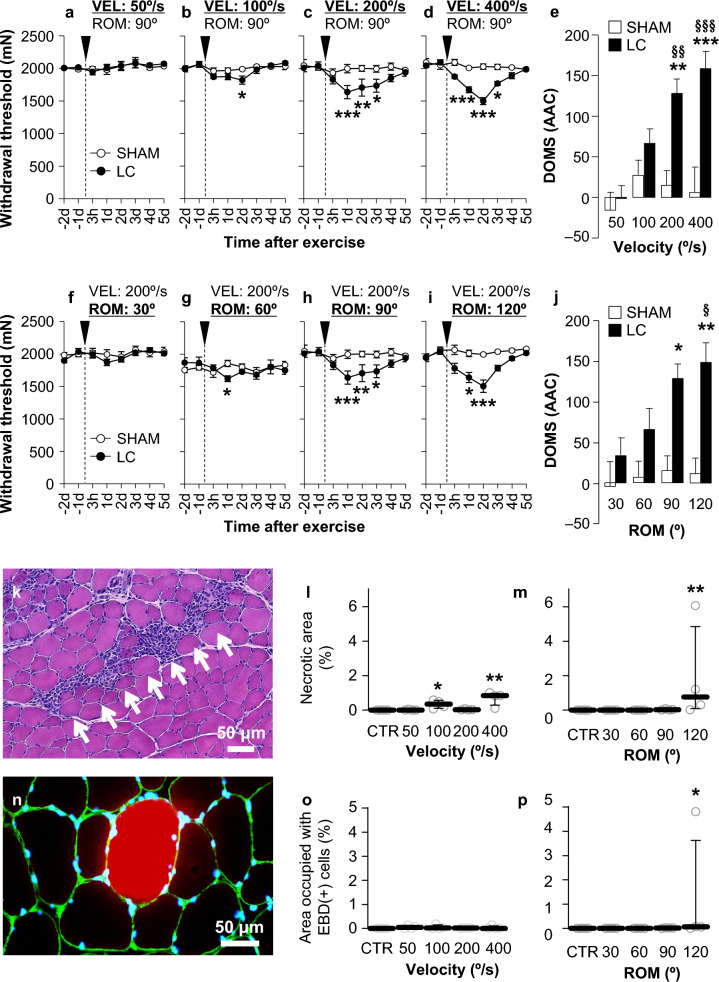
Muscular mechanical hyperalgesia after lengthening contractions in rats depends on stretch velocity (VEL) (**a**–**e**) and range of motion (ROM) (**f**–**j**), and muscle fiber damage such as necrotic fibers (**k**) and Evans Blue dye-positive fibers seldom observed (**k**–**p**). *AAC* area above the curve. Modified from Hayashi et al. [89]

Although many studies have reported that muscles are damaged after LC, which is believed to be the cause of DOMS and other changes after LC, our DOMS model showed neither injured muscle fibers nor inflammatory cell infiltration into the muscle fiber bundle [37, 87, 88]. The belief in microdamage of muscle fibers and subsequent inflammation as a cause for DOMS is very strong; we thought it was essential to find out something to solve this discrepancy and to bind current beliefs and our observations. We believe that exercise intensity might be the key to bridging this gap and quantitatively examining the relationship between DOMS and tissue damage at various exercise intensities. We introduced a machine that can precisely control stretching speed and range of motion [69] and examined the MMWT and histology of the exercised muscle. Electrical stimulation of the common peroneal nerve was performed using a previously described [78]. The range of motion and stretch velocity were varied, stimulating the nerve with a constant current strength (3 times the twitch threshold, which is considered to activate all A-fibers) to induce contraction [89]. When the range of motion was fixed at 90°, the magnitude of mechanical hyperalgesia (decrease in MMWT) correlated with the stretch velocity in the range of 100–400°/s (Fig. 3a–d), with a significant increase in the area above the curve over 200°/s (Fig. 3e). When the range of motion was fixed at a higher level (120°), a significant increase in the area above the curve was detected at a lower stretch velocity (100°/s) [89]. When the stretch speed was fixed at 200°/s, the magnitude of mechanical hyperalgesia correlated with the range of motion in the range of 60–120°, with a significant increase in the area above the curve over 90° (Fig. 3f–j). Even when the stretch velocity was fixed at a higher level (400°/s), no significant increase was observed for a smaller range of motion [89]. Histological examination revealed that the necrotic area (Fig. 3k) and the area occupied with Evans Blue-positive fibers (Fig. 3n, indication of disrupted integrity of the muscle cell membrane) were minimal in all ranges of stretch velocity and range of motion, except for the highest values (Fig. 3l, m, o, p). These results show that mechanical hyperalgesia (DOMS) is induced without muscle fiber damage, and that muscle fiber damage at the light microscopic level can be induced only when the exercise parameters are very high.

In a previous study involving rats [69], using the same stretching machine and method used in the experiment by Hayashi et al. [89] except for the use of direct electrical stimulation of the muscle instead of motor nerve stimulation, revealed substantial areas displaying Evans Blue infiltration into muscle fibers. This infiltration is indicative of muscle fiber damage, a finding that contrasts significantly with Hayashi’s results. The extent of this damage was found to vary depending on the angular velocity (range of motion fixed at 90°). Consistent with animal experiments, Crameri et al. reported infrequent observations of muscle damage following lengthening contractions (LC) with voluntary contraction, whereas damage was evident after contraction induced by electrical stimulation of the muscle [72]. In contrast, Lauritzen et al. reported that the most severe decrease in muscle power after maximal eccentric contraction, although voluntary, resulted in severe muscle damage [73]. In summary, whether muscle damage occurs depends on the strength of the LC, even with voluntary contraction, and the LC with electrical stimulation of the muscle is more prone to damage to the muscle fibers.

### B2 bradykinin receptor-NGF route

Many substances are released from exercising muscle: lactate [90]; bradykinin (including kallidin-like peptide) [91, 92]; ATP [93]; several inflammatory cytokines, such as TNF-α, interleukin (IL)-6, IL-1β and some neurotrophins have been suggested to play roles in muscular mechanical hyperalgesia [94, 95]. However, little attention has been paid to the roles of these substances in DOMS and few pharmacological manipulations have been performed, except for the use of nonsteroidal anti-inflammatory drugs [68]. Therefore, we focused on the role of bradykinin in DOMS, with special attention to the time points of its involvement.

**Fig. 4:**
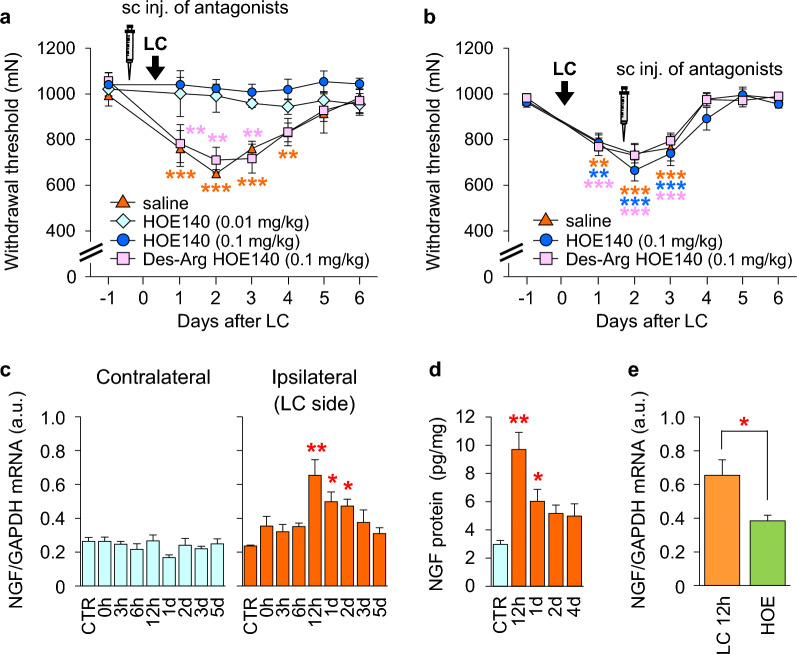
Effect of B2 bradykinin receptor antagonist HOE 140 and NGF expression in the muscle. The B2 bradykinin receptor antagonist HOE 140 completely blocked the development of DOMS when subcutaneously (s.c.) injected (**a**) but had no effect when injected in the midst of DOMS 2 days after LC (**b**). NGF mRNA (**c**) and protein (**d**) levels in exercised muscle increased 12 h–1 or 2 days after LC and were blocked by HOE 140 (**e**). Modified from Murase et al. [37]

When a B2 bradykinin receptor antagonist, HOE 140 (0.01 and 0.1 mg/kg), was subcutaneously injected into experimental animals (rats) before LC, the MMWT did not change (i.e., mechanical hyperalgesia did not develop) (Fig. 4a). However, when HOE 140 (at a higher dose) was injected at the time point with the strongest MMWT decrease, that is, 2 days after LC, no reversal of the decreased MMWT was observed (Fig. 4b). When HOE 140 was injected 30 min after LC, no effect was observed; that is, DOMS developed as usual [37]. The B1 bradykinin receptor antagonist des-Arg HOE 140 (0.1 mg/kg) had no effect when injected before or 2 days after LC (Fig. 4a, b). These results suggest that the B2 receptor agonist (bradykinin or kallidin) works in a short time window, namely, from LC to up to 30 min after LC, but no later than this; that is, a process leading to mechanical hyperalgesia was initiated by B2 bradykinin receptor activation during LC to 30 min after LC, but the hyperalgesic state was not maintained by B2 bradykinin receptor activation. The release of bradykinin during exercise has been previously reported [91, 92, 96, 97]; this bradykinin was reported to be mostly a kallidin-like peptide (Arg-bradykinin) in rats, released from blood vessels by adenosine [92]. Since HOE 140 does not differentially antagonize bradykinin, kallidin, kallidin-like peptide, from now on we use ‘bradykinin’ to refer to all three of these kinins.

We identified a substance that functions downstream of bradykinin and plays a role in maintaining mechanical hyperalgesia. NGF is known to induce muscular mechanical hyperalgesia in humans [53, 98]. We measured NGF expression in exercised muscles at various time points (Fig. 4c) after LC. The first significant increase in NGF messenger ribonucleic acid (mRNA) was detected 12 h after LC, and this increase lasted for up to 2 days after LC (Fig. 4c). Notably, there was a delay of up to 6–12 h before the upregulation of NGF mRNA was detected. This was also confirmed at the protein level (Fig. 4d). In accordance with the finding that HOE 140 injection before LC suppressed the development of mechanical hyperalgesia, HOE 140 administration before LC also suppressed the upregulation of NGF 12 h after LC (Fig. 4e). Shortening contraction or stretching neither decreased the MMWT nor upregulated NGF mRNA levels in the muscle 12 h after LC [37]. Murase et al. also investigated the possible contributions of IL-1β, IL-6, and TNF-α, none of which were suitable for a substance that works downstream of bradykinin after LC [37]. Recently, NGF upregulation in biopsy specimens was reported 24 h after LC in humans [99]. To our knowledge, this is the first report of changes in NGF expression after exercise in humans.

**Fig. 5:**
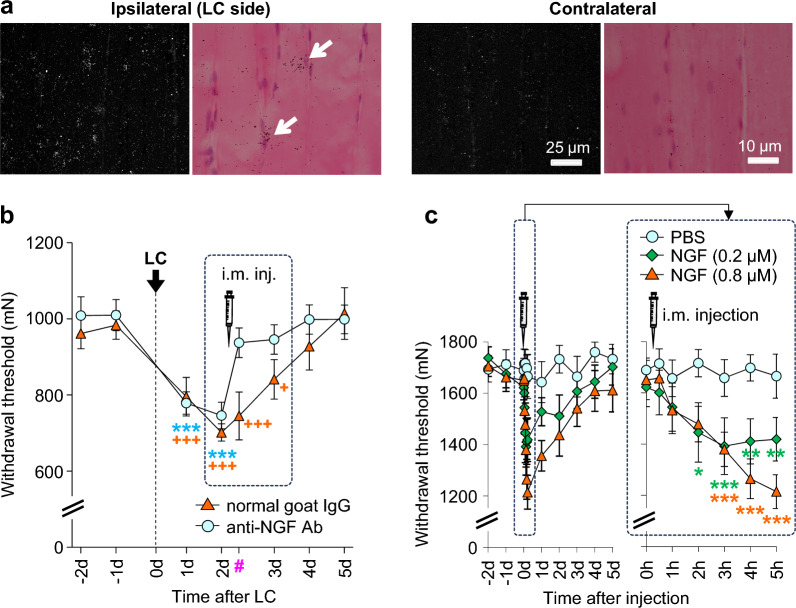
B2 bradykinin receptor–NGF route in DOMS. **a**: Dark-field (left) and bright-field (right) photomicrographs of in situ hybridization on the LC side (left pair) and contralateral side (right pair). White dots in bright-field photos and black dots in bright-field photos represent signals of in situ hybridization of NGF mRNA. Signals were observed around myofiber/satellite cell nuclei in LC side. **b**: Injection of anti-NGF antibody (30 μg) into the muscle at the time point marked with a symbol of injector almost completely reversed the decreased withdrawal threshold in 3 h (#). **c**: NGF dose-dependently decreased the withdrawal threshold 2 or 3 h after injection. Comparison was made with – 1 day. Modified from Murase et al. [37]

NGF is produced by inflammatory cells such as macrophages and mast cells [100]. However, these cells could not be responsible for NGF production after LC because they did not infiltrate the exercised muscle in our model [87]. In situ hybridization histochemistry revealed NGF mRNA signals in the periphery around the nuclei of skeletal muscles and/or satellite cells (left panels in Fig. 5a) [101]. This result shows, for the first time, that NGF is produced by muscle and/or satellite cells.

To ascertain whether NGF is responsible for mechanical hyperalgesia after LC, anti-NGF antibody was injected into the muscle after MMWT measurement 2 days after LC. With this injection, the decreased MMWT was almost completely reversed 3 h after the injection (Fig. 5b) and the effect was still observed the following day. An anti-NGF antibody injected shortly after LC completely blocked the development of mechanical hyperalgesia [37].

**Fig. 6:**
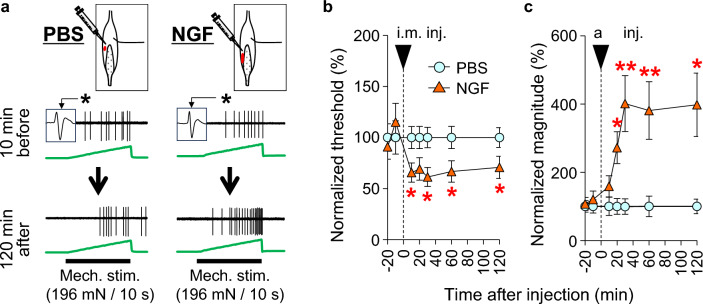
NGF-induced mechanical sensitization of muscular thin-fiber receptors. Single-fiber recordings were performed from the common peroneal nerve-extensor digitorum longus muscle preparation ex vivo. **a**: Sample recordings from C-fibers before and 120 min after intramuscular injection of PBS (left) and NGF (right) near the receptive field. The mechanical response of the fibers that received the NGF injection increased 120 min after NGF injection. **b**: Time course of the change in the response threshold to ramp mechanical stimulation after injection. **c**: Time course of the change in the response magnitude (no. of discharges induced by ramp mechanical stimulation) after the injection. * Compared with the PBS group at each time point. Modified from Murase et al. [37]

The time course of the effects of NGF on nociception was studied using behavioral tests and single-fiber recordings ex vivo [83]. NGF 0.2 μM (20 μL, i.m.) decreased the MMWT 2–5 h after injection. A higher dose (0.8 μM) decreased the MMWT at 3 h and up to 2 days after injection **(**Fig. 5c**)**. Single fiber recording from muscle thin fiber afferents (mainly C-fibers) showed NGF (0.8 μM, 5 μL) decreased the response threshold to ramp pressure stimulation 10 min after injection until observation was closed (Fig. 6a, b). The response magnitude (number of discharges elicited) increased 20 min after injection, and this effect lasted until the end of the observation period (Fig. 6a, c). NGF is known to induce hyperalgesia in a short period by peripherally sensitizing nociceptors [102, 103] and, later, by changing the expression of neurotransmitters/modulators and ion channels in the DRGs [103–108]. The quick reversal of LC-induced mechanical hyperalgesia 3–4 h after antibody injection and the quick sensitization of muscular nociceptor responses to mechanical stimulation by NGF ex vivo in 10–20 min suggest that NGF sensitizes nociceptors to mechanical stimulation peripherally without processes in the DRGs. A report using antagonists against TRP and acid-sensing ion channels [87] suggested that these peripheral effects might be induced through these ion channels.

In addition, the following mechanism is involved in the later phase (1 day or later after LC) of DOMS: receptors/channels are upregulated by NGF transported to DRGs and then orthogradely transported to the central and peripheral afferent terminals, where they play a role in the sensitization of nociceptors. Central sensitization has been suggested for DOMS in humans [109]. In other models, the descending facilitatory system [110], N-methyl-D-aspartate, and substance P receptors [111] have been suggested to be involved. Similar mechanisms may be involved in the development of DOMS.

### Cyclooxygenase (COX)-2-GDNF route

Some studies have found that non-steroidal anti-inflammatory drugs can suppress DOMS in human subjects treated before (prophylactic) and often after exercise [112, 113]; however, studies on humans are often limited to the subjective evaluation of the soreness level. As is well known, cyclooxygenase (COX)-2 is produced by mast cells and macrophages during inflammation [114]. However, in our model, neither inflammatory cell infiltration nor necrotic muscle cells were found [87]. Stretching of cultured myoblasts or myocytes induces COX-2 shortly after stretching, leading to cell proliferation and growth [115, 116]. As discussed in the previous section, it is essential to examine the effects of a substance at various time points. Therefore, in this section, the effects of non-steroidal anti-inflammatory drugs (and prostaglandins) on DOMS were studied at different time points. The COX-1 inhibitors, SC560 (10 mg/kg, p.o.) and ketorolac (10 mg/kg, p.o.), both failed to stop the development of DOMS after oral administration before LC. They also failed to reverse the decreased MMWT in the midst of DOMS 2 days after LC. In contrast, the COX-2 inhibitors celecoxib (10 mg/kg) and zaltoprofen (5–10 mg/kg) completely blocked the development of DOMS when applied before LC, but failed to reverse the developed DOMS when applied before MMWT measurement 2 days after LC [88]. We examined whether COX-2 inhibitors affected mRNA expression of NGF, IL-6 and TNF-α, which increased 12 h after LC (described in the previous section). COX-2 mRNA and protein in the exercised EDL significantly increased immediately after LC and remained upregulated up to 12 h after LC [88]. Cells producing COX-2 are identified to be muscle/satellite cells and vascular smooth muscle cells by in situ hybridization (specimens taken from the muscle immediately after LC). These data suggest that COX-2 (that produces prostaglandins) functions during and immediately (0 h) after exercise as a trigger for the development of DOMS. It must be noted that COX-2 mRNA upregulation immediately (0 h) after exercise was observed in all forms of exercise, LC, shortening contraction, and stretching; however, a significant increase in COX-2 mRNA 12 h after exercise was observed only after LC. Therefore, COX-2 upregulation immediately after LC is considered non-specific or has other functions. Thus, COX-2 upregulation at a later time point (12 h after LC) is important for the development of DOMS.

**Fig. 7:**
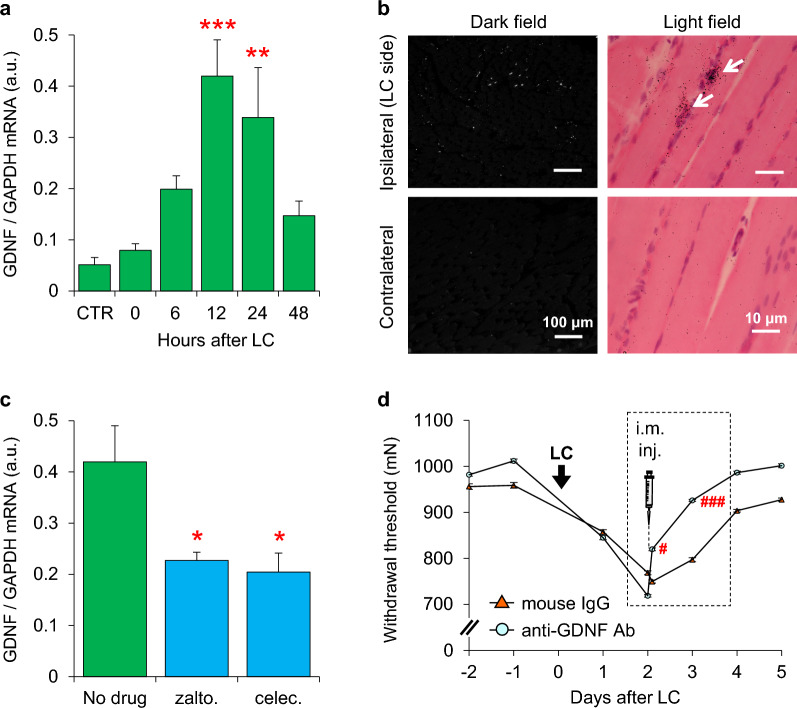
Involvement of GDNF in DOMS. **a**: Time course of change in GDNF mRNA expression in exercised muscle, * compared with the control (CTR). **b**: Photomicrograph of the in situ hybridization of GDNF mRNA. Dark field pictures (left) were taken from transverse sections at low magnification to show the mRNA signals (white dots) over a wide area. Bright field pictures (right) were taken from longitudinal sections to show the relationship between signals (black dots) and muscle cells. White arrows in bright fields photos indicate dense signals around the nuclei of muscle/satellite cells. **c**: GDNF mRNA upregulation was suppressed by COX-2 inhibitors (p.o.) prior to LC. * Compared to no drug treatment. **d**: Intramuscular injection of anti-GDNF antibody (10 μg) injected 2 days after LC reversed decreased withdrawal threshold. * Compared to pre-injection 2 days after LC. Modified from Murase et al. [88]

Similar to the B2 bradykinin antagonist, COX-2 inhibitors had no effect when administered when DOMS was fully developed. Therefore, it is natural to think that muscular mechanical hyperalgesia (DOMS) was maintained (or nociceptors were sensitized) not by prostaglandins, but by some other mediator(s). GDNF is known to be produced by muscle cells [117], is upregulated in the muscles of patients with polymyositis, Duchenne-type muscle dystrophy, and other neuromuscular diseases, and is considered a mediator of muscle pain in these pathological conditions. Therefore, we examined the role of GDNF in DOMS development. GDNF mRNA in exercised EDL significantly increased 12 h–1 day after LC (Fig. 7a), later than the increase of COX-2 mRNA, but increased neither after shortening contraction nor stretching. Other GDNF family ligands, such as artemin, neurturin, and persephin, did not increase after LC [88]. The signals of GDNF mRNA visualized with in situ hybridization in the ipsilateral EDL muscle 12 h after LC increased compared to those in the contralateral side without LC (Fig. 7b). Signals were mostly observed around the cell nuclei. We could not identify whether these nuclei were from skeletal muscle or satellite cells. Both COX-2 inhibitors suppressed GDNF mRNA upregulation 12 h after LC (Fig. 7c). To confirm the involvement of GDNF in DOMS, an anti-GDNF antibody (10 μg) was intramuscularly injected 2 days after LC, which significantly reversed the decrease in MMWT at 3 h and later after injection (Fig. 7d).

**Fig. 8:**
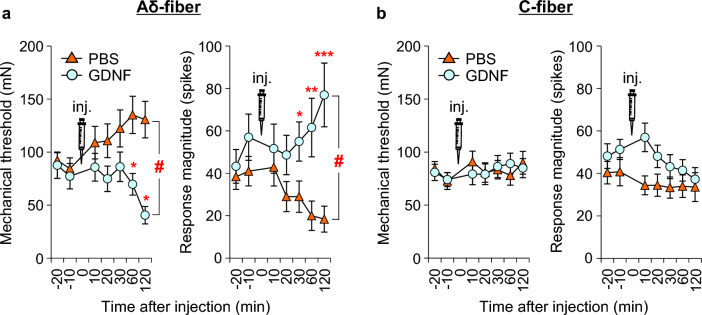
GDNF-induced mechanical sensitization occurs in muscle Aδ-fibers. **a**: Response threshold (left) and response magnitude (right) to a ramp mechanical stimulation from 0 to 196 mN in 10 s in Aδ-fibers (*n* = 14 each). Significant changes were observed after 60 (threshold) and 30 min (magnitude). **b**: Those of C-fibers (*n* = 30 each). No changes were observed in either the mechanical threshold or response magnitude. Modified from Murase et al. [38]

**Fig. 9:**
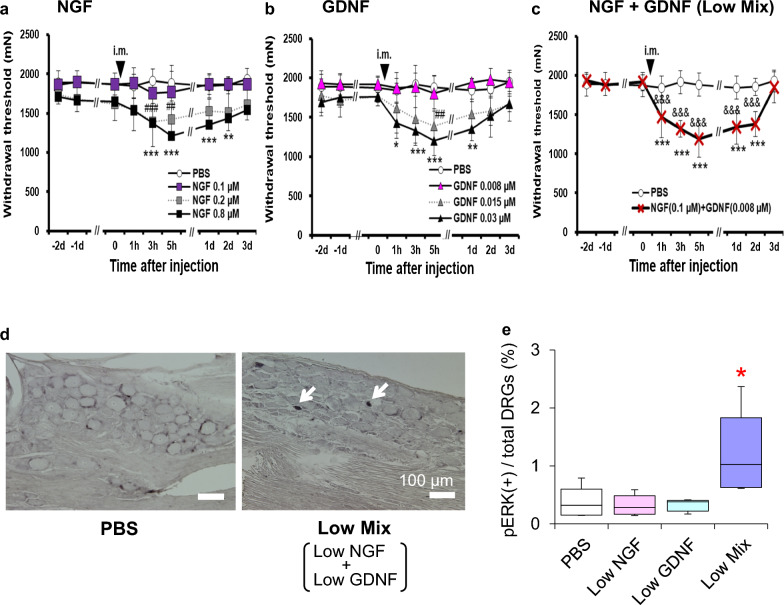
NGF and GDNF synergistically amplifies muscular hypersensitivity. **a**: Dose–response relationship of the NGF-induced decrease of MMWT (mechanical hypersensitivity). *n* = 6 except 0.2 μM and 0.8 μM NGF (*n* = 8). **b**: Dose–response relationship of the GDNF-induced decrease in MMWT. *n* = 6 each. **c**: A mixture of NGF (0.1 μM) and GDNF (0.008 M), which alone did not induce a decrease in MMWT, induces a pronounced decrease in MMWT. *n* = 6. **d**: Sample photograph of pERK immunohistochemistry of the DRG (L5) after compression (1500 mN) of the muscle treated with PBS (left) or a mixture of low NGF (0.1 μM) and low GDNF (0.008 μM) (right). **e**: Percentage of pERK^+^ DRG neurons after each treatment. Neither low NGF nor low GDNF induced a larger percentage of pERK^+^ neurons compared with PBS, but a mixture of both (Low Mix) induced a significantly larger increase. Modified from Murase et al. [19]

Single-fiber recordings from muscle nerve preparations ex vivo showed that, unlike NGF, C-fiber afferents were not sensitized by GDNF (0.03 μM) for up to 2 h after injection (Fig. 8b). Instead, Aδ-fibers were sensitized 1 h after injection, and remained sensitized up to 2 h after injection (Fig. 8a) [38]. The presently observed Aδ-fiber involvement in DOMS is somewhat unexpected in that DOMS is a dull pain. It is believed that Aδ-fibers transmit pricking sensation (fast pain), and C-fibers are responsible for burning sensation (slow pain) in the skin. However, intraneural microstimulation of the muscular Aδ- and C-afferent fibers revealed that both fibers induced the same dull, aching, or cramping sensation [118]. Muscular mechanical hyperalgesia was dose dependently induced by intramuscular injection of GDNF (0.008–0.03 μM, 20 μL) [19, 38]. A decrease in the MMWT first appeared 1 h after injection and lasted up to the next day at a higher dose (Fig. 9b) [38].

### Interaction between NGF and GDNF routes

As described in the previous two sections, two routes are involved in the development of DOMS: the B2 bradykinin receptor activation-NGF route and the COX-2-GDNF route. COX-2 inhibitors did not suppress NGF upregulation; however, the B2 receptor antagonist HOE 140 suppressed COX-2 and GDNF upregulation 12 h after LC. The induction of COX-2 by B2 bradykinin receptor upregulation is known to occur in DRG neurons [119], airway epithelial cells, and arterial smooth muscle cells [120]. Thus, interactions occur between the two routes at the level of COX-2 production.

**Fig. 10:**
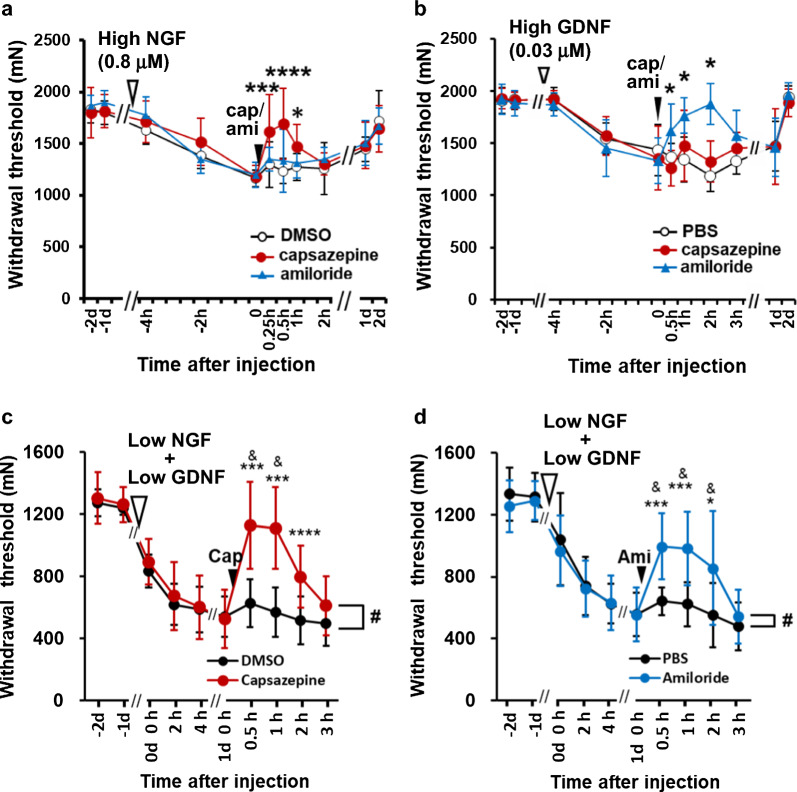
Involvement of TRPV1 and ASICs in NGF- and GDNF-induced hypersensitivity. High NGF (0.8 μM, 20 μL, i.m.)-induced decrease in MMWT was reversed by capsazepine (50 μM, 20 μL) but not by amiloride (50 mM, 20 μL) (**a**), whereas high GDNF (0.03 μM)-induced decrease in MMWT was reversed by amiloride but not capsazepine (**b**), suggesting that TRPV1 is involved in high NGF-induced hypersensitivity, whereas ASICs are involved in high GDNF-induced hypersensitivity. A mixture of low NGF- and low-GDNF-induced decrease in MMWT was reversed by both capsazepine (**c**) and amiloride (**d**), suggesting that both receptor channels are involved in low mixture-induced hypersensitivity. Mean ± SD. **a**, **c**, and **d**: Modified from Murase et al. [19]; **b**: Modified from Murase et al. [38]

Another significant interaction is evident from the fact that the application of a COX-2 inhibitor before lengthening contractions (LC) leads to the complete suppression of delayed-onset muscle soreness (DOMS), despite the unaffected status of NGF upregulation [88]. Additionally, a notable observation arises from the combined application of NGF and GDNF at concentrations that, when applied individually, do not induce a decrease in MMWT (NGF 0.1 µM; GDNF 0.008 µM). This combination, however, results in a pronounced MMWT decrease comparable to that induced by concentrations 4–8 times higher (Fig. 9a–c). This effect cannot be considered simply an addition of the effects of two substances at the subthreshold level because receptors for both substances are reported to be expressed in different sets of DRG neurons [121] and use different receptor ion channels (NGF: TRPV1, GDNF: ASIC3) (Fig. 10a, b). We hypothesized that the site of interaction was the primary afferent nerve. To clarify this, the expression of phosphorylated extracellular signal-regulated kinase (pERK), which has been used as a marker of neuronal activation [122], was examined after compression of the muscle that received neurotrophic factors (thus, the sensitizing effects of NGF and GDNF were examined). Compression after NGF or GDNF alone at low concentration (0.1 and 0.008 μM, respectively) did not increase pERK immunoreactivity compared to injection of phosphate buffered saline (PBS). When a mixture of low NGF and GDNF levels was injected, pERK immunoreactivity increased significantly after mechanical stimulation (Fig. 9d, e). This observation indicated that the interaction between NGF and GDNF occurred at the primary afferent level. The effects of high NGF or GDNF levels were blocked by either TRPV1 specific antagonist capsazepine or the ASICs inhibitor amiloride, but not by another (Fig. 10a, b). In contrast, the decrease in the MMWT induced by the combination of low levels of NGF and GDNF was reversed by capsazepine and amiloride (Fig. 10c, d). It has been reported that TrkA activation by NGF promotes RET (receptor for GDNF) phosphorylation in ligand-independent manner in mature sympathetic neurons [123]. If this also occurs in DRG neurons and both A-fiber and C-fiber neurons express both GDNF and NGF receptors at different levels, then the above-described inhibition of low-mixture-induced hyperalgesia by capsazepine and amiloride could be explained. Double expression of the NGF and GDNF receptors in DRG neurons is discussed in the following section.

NGF specifically binds to tropomyosin-related kinase A (TrkA) [124] with high affinity and GDNF interacts with a receptor complex consisting of RET receptor and a GFRα1 coreceptor [125]. GFRα1 serves as a ligand-binding domain that has no intracellular domain and is anchored to the cell membrane with glycosylphosphatidylinositol, and the RET receptor that has no ligand binding domain and serves as a signal-transducing domain with tyrosine kinase activity. Primary sensory neurons expressing TrkA in adult animals, and those expressing GFRα1 are believed to be different sets of afferents [121]. For both neurotrophins to interact at the primary afferent level, both receptors (receptor complexes) must be co-expressed in DRG neurons. Since a great majority of DRG neurons are skin innervating, TrkA expression and GFRα1/RET expression in DRG neurons innervating the muscle might be different from that of cutaneous or whole DRG neurons (majority is cutaneous) as reported by Priestley et al. [121]. This point was examined by retrograde tracing from gastrocnemius muscle (GC) with Fluorogold (FG) and double in situ hybridization of TrkA and GFRα1 mRNA, and it revealed that 23.7–29.2% of GC-innervating DRG neurons co-expressed TrkA and GFRα1 (Fig. 1) [19]. The cell size of the co-expressing neurons (shown in yellow in Fig. 1) was distributed widely from small to large; therefore, small-to medium-sized co-expressing neurons (8–15% of DRG neurons innervating the GC), which are thought to have thin axons and play roles in nociception, are thought to contribute to this mechanical hyperalgesia. However, the role of large DRG neurons expressing both receptors remains unclear.

**Fig. 11:**
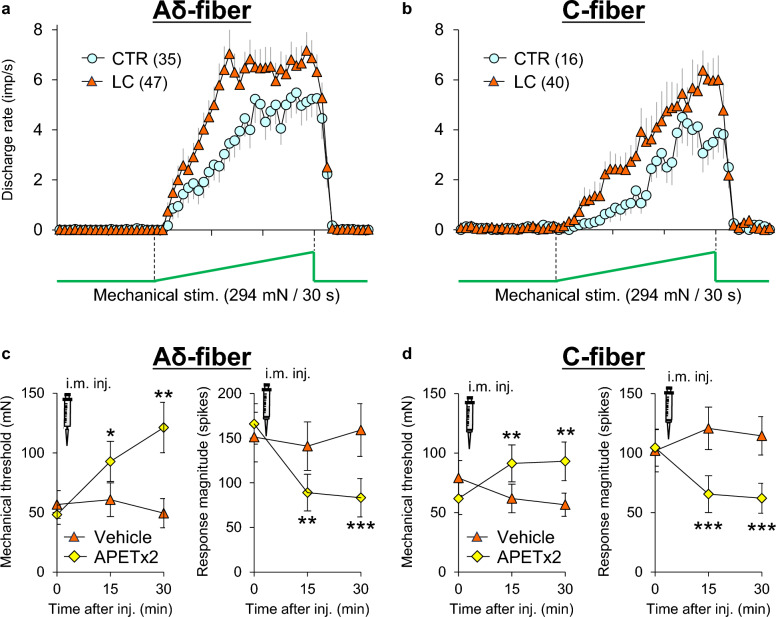
Mechanical sensitization of thin-fiber receptors after LC is mediated by ASIC3. Single-fiber recordings from Aδ- and C-fibers were performed from the common peroneal nerve-extensor digitorum longus muscle preparation ex vivo. Average time histograms of the mechanical responses of Aδ- (**a**) and C-fibers (**b**) from control rats and rats that underwent LC. Thus, the response of the LC group was facilitated. The ASIC3 inhibitor APETx2 (2.2 μM) injected near the receptive field reversed the decreased mechanical threshold (left) and increased the response magnitude (right) of both Aδ- (**c**) and C-fibers (**d**). Modified from Matsubara et al. [126]

Since mechanical hyperalgesia after LC was reversed by both capsazepine and amiloride [87], similar to hyperalgesia after low NGF and GDNF mixture injection, concentrations of NGF and GDNF in the DOMS muscle might not be as high as high doses used by Murase et al. [37, 38], and rather low as low doses used by Murase et al. [19]. In concert with this observation, APETx2, a specific inhibitor of ASIC3, reversed the sensitized mechanical responses of both Aδ- and C-fibers after LC (Fig. 11) [126].

To elucidate the distinct involvement of ion channels and afferent fiber classes—Aδ-fibers for GDNF and predominantly C-fibers for NGF, as detailed in the previous section, in high NGF- and high GDNF-induced mechanical hyperalgesia—one must consider neurons expressing both TrkA and GFRα1/RET, hypothesizing that they express both TRPV1 and ASICs. However, the relative expression levels remain variable. It is conceivable that one subset of neurons, potentially C-fiber neurons, expresses higher levels of TrkA and TRPV1 compared to GFRα1/RET and ASICs. Conversely, another subset, possibly Aδ-fiber neurons, might express elevated levels of GFRα1/RET and ASICs relative to TrkA and TRPV1. Regrettably, current knowledge lacks information on these specific points. Furthermore, the precise intracellular signaling mediating the interaction between the TrkA and RET systems remains unknown. These unresolved aspects underscore the necessity for future studies to shed light on these crucial details.

### Ion channels involved in mechanical sensitization in DOMS

The involvement of receptor ion channels, other than TRPV1 and ASIC3, in DOMS has also been studied in mice. DOMS was induced in mice using almost the same method as in rats: the muscle used was changed to GC and the number of LC was changed 300 times. The MMWT decreased 6–36 h after LC, with earlier development and shorter duration of DOMS than in rats [86]. Corresponding to this time course, the upregulation of NGF and GDNF mRNA was observed only 3 h after LC. COX-2 upregulation was observed 0 and 3 h after LC. DOMS was induced neither in TRPV1^−/−^ mice nor TRPV4^−/−^ mice. NGF i.m. induces mechanical hyperalgesia in wild-type (WT) and TRPV4^−/−^ mice but not in TRPV1^−/−^ mice. GDNF i.m. induced mechanical hyperalgesia in WT mice but not in TRPV1^−/−^ or TRPV4^−/−^ mice. These results show that both TRPV1 and TRPV4 are involved in DOMS and GDNF-induced mechanical hyperalgesia and that only TRPV1 is involved in NGF-induced mechanical hyperalgesia.

### Schema of mechanism

**Fig. 12:**
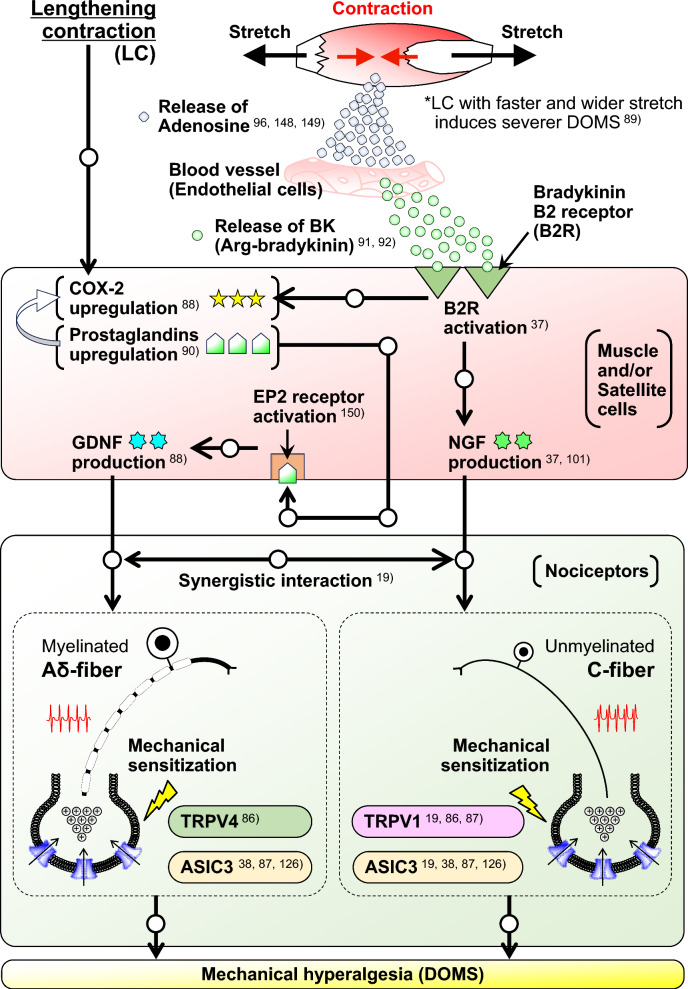
Neurochemical mechanisms for the development of DOMS. The starting point of this schema is based on the report by Boix et al. [92] that a bradykinin-like substance (Arg-bradykinin) is produced and released from blood vessels by the adenosine released by muscle contraction [148, 149]. Arg-bradykinin binds to and activates the B2 bradykinin receptor (B2R) in muscle cells to stimulate NGF production. NGF sensitizes C-fibers only when high concentrations are used, and both Aδ- and C-fibers when a low mix is used or in DOMS involving TRPV1 and ASIC3. In addition, the activation of B2R upregulates COX-2. Another route involves the upregulation of COX-2 in muscle fibers, resulting in increased production of prostaglandin (PG) E2. PGE2 rapidly spreads from the cells and binds to the EP2 receptor [150] to stimulate GDNF production. GDNF sensitizes nociceptors (Aδ-fibers only when high concentration was used, and both Aδ- and C-fibers when low mix was used or in DOMS) involving TRPV4 and ASIC3. A synergistic interaction has been observed between NGF and GDNF at the primary afferent level. The mechanism of this synergistic interaction is open to future studies

A schematic diagram summarizing the observations described in the previous sections (Sections from "B2 bradykinin receptor-NGF route" to "Ion channels involved in mechanical sensitization in DOMS") is shown in Fig. 12.

### Repeated bout effect

**Fig. 13:**
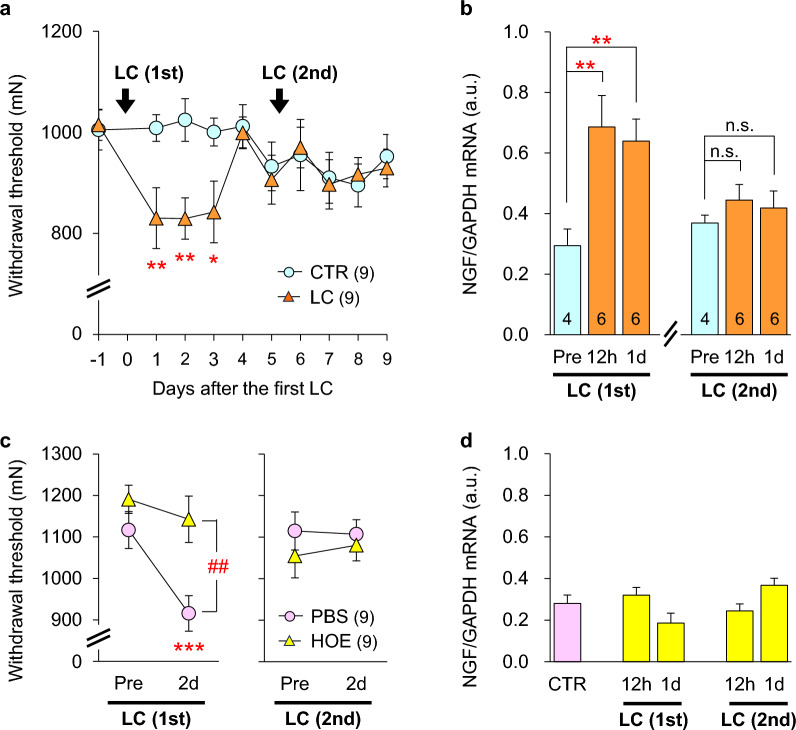
DOMS is attenuated by repeated LC. **a**: A Decrease in MMWT after LC did not occur after the second bout of LC with a 5-day-interval. **b**: NGF mRNA increased after 1st LC but not after 2nd LC, corresponding to the absence of DOMS after 2nd LC. **c**: Subcutaneous injection of the B2 receptor antagonist HOE140 before 1st LC suppressed DOMS not only after 1st LC but also after 2nd LC, although the effect of HOE 140 lasts for several hours. Consistent with this observation, NGF mRNA levels did not increase in either group (**d**). These results suggest that adaptation to LC occurs prior to B2 bradykinin receptor activation. Modified from Urai et al. [132]

Repeated bout effect, first reported by Nosaka et al. [127] is a phenomenon of soreness (Fig. 13a), swelling, decrease in voluntary muscle isometric contraction power, and other changes after LC that are less when exercise is repeated within several weeks [128], compared with the initial bout. The repeated bout effect was greater when the LC exercise in the first bout was strong (e.g., 100% maximal isometric contraction force), but it was also obtained with the 1st bout performed at 40% maximal isometric contraction force [129]. Even isometric contraction three weeks before LC (not immediately before LC) prevents the development of muscle changes [130]. A comprehensive review of repeated bout effect has been published [131], and neural adaptations, alterations to mechanical properties of the muscle–tendon complex, extracellular matrix remodeling, and biochemical signaling in repeated bout effect were introduced as the mechanism of repeated bout effect, proposing that these factors work in concert to coordinate protective adaptation. However, the possible mechanism of the repeated bout effect in DOMS has not yet been elucidated. Urai et al. [132] showed that mechanical hyperalgesia did not develop after 2nd LC 5 days after the first LC (Fig. 13a), and that NGF mRNA was not upregulated in exercised muscle after 2nd bout, corresponding to no decrease in MMWT (decrease in DOMS) (Fig. 13b). Urai et al. also examined whether this adaptation occurred before B2 receptor activation. When the B2 receptor antagonist, HOE 140, was administered before 1st LC, no decrease in MMWT was observed as expected [37]. Notably, no mechanical hyperalgesia developed after the 2nd LC in rats received B2 receptor antagonist only before the 1st bout (Fig. 13c). The NGF mRNA levels did not increase after both 1st and 2nd bouts in the group that received the B2 receptor antagonist before 1st LC (Fig. 13d). The effect of HOE 140 lasted for only several hours; therefore, the absence of DOMS and NGF mRNA upregulation after 2nd LC was not because the B2 receptors were still blocked by the antagonist. These results suggest that adaptation to LC occurred at least somewhere before B2 receptor activation, possibly production/release of bradykinin-like substances, or further upstream to this, namely, release of adenosine and/or change in endothelial cells that received mechanical stress by LC (Fig. 12). Repeated bout effect in the COX-2-GDNF route has also been reported [133]. Further experiments are required to clarify the mechanism of repeated bout effect on DOMS.

Repeated bout effect in NGF mRNA was also reported in humans [99].

### LC-induced DOMS in the human low back

**Fig. 14:**
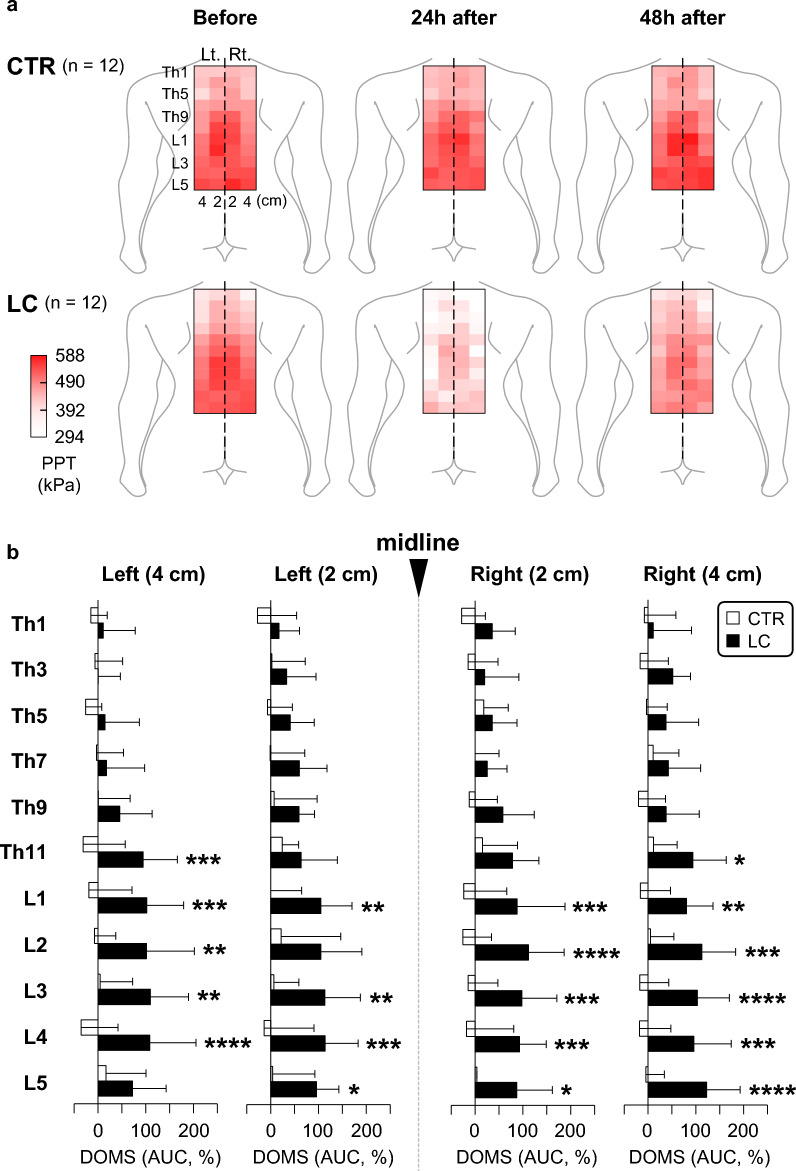
Human model of DOMS in the thoracolumbar paraspinal muscles. **a** Distribution of pressure pain thresholds. In the CTR group (*n* = 12), there were no remarkable changes in the pressure pain threshold maps at 0 (before LC), 24, and 48 h after LC. In the LC group (*n* = 12), the pressure pain thresholds remarkably decreased 24 h after LC, and the decreased threshold appeared to recover 48 h after LC. Heatmap images were obtained from the mean pressure pain threshold values at each measurement point (ranging from 294 to 588 kPa). **b**: Magnitude of DOMS in the thoracolumbar area after LC. Note the significantly higher magnitude of DOMS in the LC group than in the CTR group at segments Th11–L5 (*p < 0.05, **p < 0.01, ***p < 0.001, and ****p < 0.0001; CTR vs. LC, two-way repeated-measures analysis of variance followed by Sidak’s multiple comparison test). Modified from Hanada et al. [137]

While DOMS in extremity (limb) muscles such as knee extensors and elbow flexors has been well documented [134–136], DOMS in the lower back, which can often be induced in everyday work and athletic games, has been less characterized. To obtain topographic images of DOMS in the lower back, a repetitive LC was applied to the paraspinal muscles in the thoracolumbar area [137]. Participants had their trunk fall from a starting position (parallel to the floor) to a 40° flexed position and then returned as quickly as possible to the starting position. The LC cycle was repeated until the participants could no longer maintain their contractions. Pressure pain thresholds were systematically measured in the bilateral paraspinal muscles of the thoracolumbar area at the level of the spinous processes at Th1–L5. The measurement points were 2 and 4 cm from the midline, and the measurements were repeated before LC and 24 and 48 h after LC [137]. In control participants without LC, the pressure pain thresholds remained unchanged over time (Fig. 14a). In contrast, the pressure pain thresholds of the participants who underwent LC decreased 24 h after LC and recovered 48 h after LC (Fig. 14a). No left–right or mediolateral preference was observed for the distribution of pressure pain thresholds. However, a remarkable decrease in the pressure pain thresholds was detected in the paraspinal muscles in the lumbar segments compared to those in the thoracic segments (Fig. 14b). The topographic distribution of pressure pain thresholds may be of clinical importance in the treatment of lower back muscle pain after exercise (DOMS).

### Prevention, treatment of DOMS and usage of LC for strengthening the muscle

LC is known to increase muscle mass (even with almost the same O_2_ consumption as concentric exercises) [62], and athletes use LC to strengthen their muscles. Athletes believe that muscle must be damaged to get stronger. However, Flann et al. showed that muscle hypertrophy could be initiated independently of discernible muscle damage [138].

Our experiments [88] showed that COX-1 inhibitor treatment had neither a preventive nor therapeutic effect on DOMS, and COX-2 inhibitors had only a preventive effect. Shimodaira et al. have recently reported that aspirin, ibuprofen, loxoprofen, and acetaminophen administered 24 h after LC reversed the decreased MMWT dose-dependently [139], while they confirmed that celecoxib had no effect with the same procedure as Murase et al. [88]. Shimodaira et al. [139] suggested that the COX-independent pharmacological action of COX inhibitors could be induced by such as inhibition of TRPV1 and ASICs [140, 141] involvement of which in DOMS were reported in several studies [86, 87, 126], and inhibition of protein kinase C activity, which is involved in sensitization of TRPV1 by NGF [142].

Hot packs (thermal treatment) and massage (manual therapy) are often used for the treatment of DOMS, and their effectiveness has been reported [79, 143, 144]. The mechanisms underlying their effectiveness have been studied only a little [79].

The property that the LC strengthens muscles with lower O_2_ consumption would be beneficial for those who have problems in cardiovascular and/or respiratory system(s). However, LC induces DOMS, thus discouraging individuals from exercising. DOMS can be avoided or reduced by prior exercise with either a weaker LC [145] or isometric contraction [130]. Using these methods, LC can be used by the elderly to strengthen muscles to prevent falls or flails [65, 146].

### Summary and perspective of DOMS study

In this review, we briefly show that two routes are involved: the B2-bradykinin receptor-NGF route and the COX-2-GDNF route. Their initial mechanical events remain unclear. We also showed that the two routes interact at the primary afferent level and that TrkA and GFRα1 co-exist in small-to medium-sized DRG neurons, which enables interaction. The mechanism by which these interactions occur remains open for future studies. In addition, the repeated bout effect of DOMS was interpreted based on the B2-bradykinin receptor-NGF route, and we showed that the repeated bout effect occurred before B2 bradykinin receptor activation. The initial event of the repeated bout effect is intriguing and requires further study.

## Data Availability

Data sharing is not applicable to this article, as no datasets were generated or analyzed in the current study.

## Abbreviations

ASIC: Acid-sensing ion channel
COX: Cyclooxygenase
DOMS: Delayed onset muscle soreness
DRG: Dorsal root ganglion
EDL: Extensor digitorum longus muscle
FG: Fluorogold
GC: Gastrocnemius muscle
GDNF: Glial cell line-derived neurotrophic factor
GFRα1: GDNF family receptor α 1
i.m.: Intramuscular
LC: Lengthening contraction
MMWT: Muscular mechanical withdrawal threshold
mRNA: Messenger ribonucleic acid
NGF: Nerve growth factor
PBS: Phosphate buffered saline
pERK: Phosphorylated extracellular signal-regulated kinase
p.o.: Per os
RET: Rearranged during transfection
TNF-α: Tumor necrosis factor-α
TrkA: Tropomyosin-related kinase A
TRPV: Transient receptor potential cation channel subfamily V member
WT: Wild-type
